# The Potential for Cancer Immunotherapy in Targeting Surgery-Induced Natural Killer Cell Dysfunction

**DOI:** 10.3390/cancers11010002

**Published:** 2018-12-20

**Authors:** Marisa Market, Katherine E. Baxter, Leonard Angka, Michael A. Kennedy, Rebecca C. Auer

**Affiliations:** 1 Department of Medicine, University of Ottawa, Ottawa, ON K1H8L1, Canada; kbaxt090@uottawa.ca (K.E.B.); langk069@uottawa.ca (L.A.); 2 Ottawa Hospital Research Institute (OHRI), Ottawa, ON K1G4E3, Canada; mikennedy@ohri.ca

**Keywords:** natural killer cell, surgery, immunosuppression, cancer, metastasis, immunotherapeutics

## Abstract

Natural Killer (NK) cells are granular lymphocytes of the innate immune system that are able to recognize and kill tumor cells without undergoing clonal selection. Discovered over 40 years ago, they have since been recognized to possess both cytotoxic and cytokine-producing effector functions. Following trauma, NK cells are suppressed and their effector functions are impaired. This is especially important for cancer patients undergoing the removal of solid tumors, as surgery has shown to contribute to the development of metastasis and cancer recurrence postoperatively. We have recently shown that NK cells are critical mediators in the formation of metastasis after surgery. While research into the mechanism(s) responsible for NK cell dysfunction is ongoing, knowledge of these mechanisms will pave the way for perioperative therapeutics with the potential to improve cancer outcomes by reversing NK cell dysfunction. This review will discuss mechanisms of suppression in the postoperative environment, including hypercoagulability, suppressive soluble factors, the expansion of suppressive cell populations, and how this affects NK cell biology, including modulation of cell surface receptors, the potential for anergy, and immunosuppressive NK cell functions. This review will also outline potential immunotherapies to reverse postoperative NK dysfunction, with the goal of preventing surgery-induced metastasis.

## 1. Natural Killer Cells

Natural Killer (NK) cells are innate cytotoxic lymphocytes that play a critical role in immune homeostasis by destroying circulating stressed, infected, or transformed cells [1,2]. Mature NK cells comprise 10–15% of total peripheral blood leukocytes [3] and are described as CD3^−^ CD14^−^ CD19^−^ CD56^+^ CD16^+^. Two functional subsets of NK cells can be distinguished based on the cell surface density of CD56 and the low-affinity Fc-receptor CD16 [4,5]. CD56^dim^ CD16^+^ cells make up 90% of peripheral blood and spleen NK cells and are preferentially cytotoxic, whereas most lymph node NK cells are CD56^bright^ CD16^dim/−^ and readily produce cytokines [4,6]. Natural Killers do not undergo clonal selection, but instead express germline receptors which integrate activating and inhibitory signals to determine cellular activity. Through a phenomenon known as the “missing-self hypothesis” [7] NK cells are inhibited by the recognition of constitutively expressed self-molecules. Specifically, inhibitory Killer Ig-like receptors (KIRs) and NKG2A-CD94 recognize HLA-class I and HLA-E, respectively [6,8]. Tumour cells downregulate HLA-class I in an attempt to escape T cell mediated responses [9], simultaneously releasing this inhibitory “break” on NK cells. These inhibitory signals are antagonized by activating receptors, which function by the “induced-self recognition model” [7,10,11,12]. Natural Cytotoxicity Receptors (NCRs), NKp30/44/46, and NKG2D are activating receptors that bind stress ligands BAT3, MICA/B, and ULBP1-6 [5,8], which are upregulated in tumor cells [13]. NK cells are therefore perfectly positioned to target tumor cells without immunological priming. NK cell development and activity are also mediated by contact-dependent interactions with other immune cells, including macrophages and dendritic cells, as well as a plethora of soluble factors, such as interleukin (IL)-2, IL-12, IL-15, and IL-18 [14,15,16,17,18,19,20]. Upon recognition and activation by a tumor cell, NK cells respond in three ways: (1) They are directly cytotoxic through Ca^2+^-dependent exocytosis of cytolytic granules, (2) they secrete cytokines with both direct anti-tumor and immunomodulatory properties, and (3) they induce apoptosis through the expression of death receptor ligands [5,7,13,21,22]. NK cells also participate in antibody-dependent cell-mediated cytotoxicity (ADCC) using the Fc receptor CD16 [23].

## 2. Dysfunctional NK Cells Mediate Metastasis in the Postoperative Period

**Figure 1:**
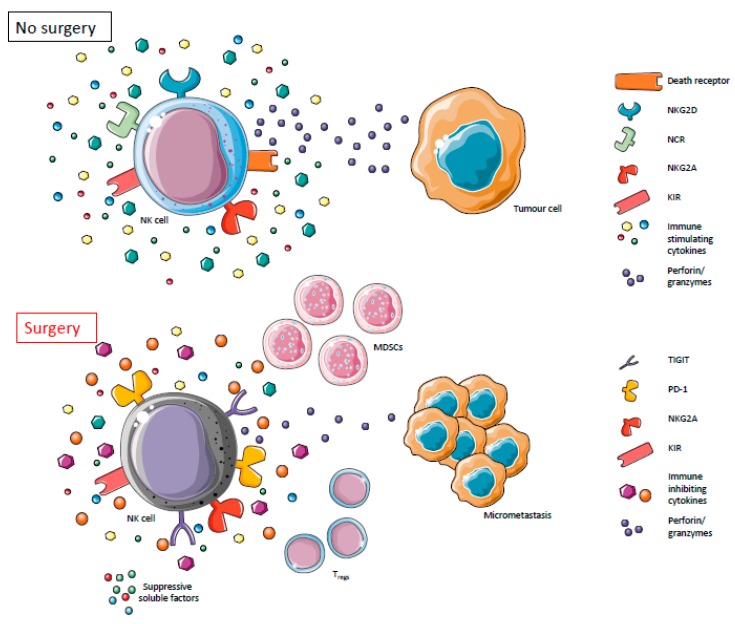
Natural Killer (NK) cells effector functions are impaired postoperatively. In the absence of surgery, NK cells are able to appropriately exert their effector functions thereby directly and indirectly acting to suppress tumor growth and proliferation. In the postoperative period, however, NK cell function is suppressed, leading to a metastatic formation in cancer surgery patients. In our proposed model, surgically stressed NK cells undergo changes in receptor expression mediated by stress-induced soluble factors and surgery-induced suppressive cell populations.

Natural killer cells are critical innate immune cells and as such, their dysfunction has significant biological consequences. The impairment of NK cell effector functions has been recorded in response to physical trauma, including thermal injury [24], spinal cord and brain injury [25,26], surgery [27,28,29,30,31], and in critically ill patients [32]. The magnitude and duration of NK cell dysfunction are determined by the extent of the injury and although this dysfunction is relatively short-lived, there are long-lasting consequences for patients, most notably in terms of infection and recovery rates [30,33]. Immune dysfunction after surgery is even more consequential in cancer patients. Evidence suggests that surgery for the resection of solid tumors may increase rates of disease recurrence, metastasis, and death [34,35,36]. This relationship between tumor resection and metastasis was first observed in 1913, however only recently has the dysfunction of the cellular immune response been implicated in the regulation of this phenomenon [27,30,37,38,39,40]. Specifically, NK cell dysfunction is thought to play an essential role in postoperative e metastasis and cancer recurrence. Profound suppression of NK cell cytotoxicity and cytokine secretion, specifically IFNγ, has been observed on postoperative day (POD) 1 and beyond, and has been correlated with increased metastases in numerous animal models of spontaneous and implanted metastases. Postoperative NK cell dysfunction is also associated with higher rates of cancer recurrence in human patients [27,28,29,30,41,42,43,44,45] (Figure 1). Historically, the perioperative administration of immunostimulatory cytokines, such as IL-2 [46], GM-CSF [47,48], TNFα [49], and IFNα [47], have shown promising results in reducing cancer recurrence and metastases. These factors are known to stimulate NK cell activity and therefore, in light of recent studies that highlight the role of NK cell dysfunction in postoperative metastatic formation, the mechanisms of these therapies are more clearly understood.

The mechanism(s) responsible for postoperative NK cell dysfunction are complex and currently incompletely characterized. Knowledge of these mechanisms will pave the way for the development of perioperative immunotherapeutics; an unexploited window of opportunity to reduce metastasis and cancer recurrence in surgical oncology patients [36]. This review will discuss in detail mechanisms of NK cell suppression in the postoperative environment and the impact on NK cell biology, and will outline potential immunotherapies to reverse postoperative NK cell dysfunction.

## 3. The Postoperative Environment and Potential Therapeutics

### 3.1. The Hypercoagulable State

Surgical stress results in a dynamic postoperative environment characterized by hypercoagulability, the release of anti-inflammatory factors, and the expansion of immunosuppressive cell populations that may be responsible for the induction of NK cell dysfunction.

The relationship between the coagulation system and cancer progression has been well characterized. Dysregulation of matrix metalloproteases promotes tumor cell migration and metastasis as a result of increased tissue factor, fibrin, and thrombin. Furthermore, angiogenesis is induced by plasmin and vascular endothelial growth factor (VEGF), which can be released from activated platelets, as well as tumor cells [50,51,52,53,54,55,56]. Moreover, surgery and cancer, independently, induce a hypercoagulable state characterized by fibrin and platelet clots around tumor cells, resulting in tumor cell emboli (TCE) that impair NK cell-mediated tumor clearance [57]. Lison et al. studied the timing of this dysfunction and found that most clotting factors were profoundly reduced on POD1 until POD3, while fibrinogen, factor VIII, and von Willebrand factor increased continuously starting on POD2, although no significant change was observed in global coagulation tests (prothrombin time (PT) and activated partial thromboplastin time (aPTT)) [58]. Furthermore, Ulrych et al. found that this hypercoagulation lasted until 28–30 days postoperatively in patients undergoing surgery for benign disease [59]. The mechanism of robust adhesion of platelets to tumor cells is largely dependent on the expression of P-selectin (CD62P) on activated platelets [60,61,62]. Cuiling et al. showed that deletion of P-selectin suppressed intestinal tumor growth in spontaneous intestinal tumor mouse models [62]. Interestingly, they also described a mechanism whereby platelet adhesion to tumor cells induces angiogenesis through the secretion of VEGF [60]. Seth et al. investigated metastasis in a murine model of surgical stress and found that surgical stress increased pulmonary metastasis and promoted coagulation [57]. Furthermore, these effects were not mouse-strain or cell-type specific. They found that both factor Xa (the activated form of thrombokinase, which cleaves prothrombin to thrombin) and P-selectin increased postoperatively and mirror the pro-metastatic timeline of surgery. To determine the mechanism of increased metastasis they assessed the association of fibrin and platelet clots with TCE in the lungs of surgically stressed mice and found a 2-fold increase in platelet clot formation and a 3-fold increase in the percent of TCE associated with fibrin when compared with no-surgery mice [57]. They tested five anti-coagulants with different mechanisms of action (tinzaparin, deltaparin, hirudin, warfarin, and platelet depletion using α-platelet GPIbα). All perioperative anticoagulants attenuated the formation of these clots in surgically stressed mice [57]. However, the increased association of fibrin and platelet clots with TCE was not observed when mice were pre-treated with heparin, a P-selectin inhibitor. In addition, to investigate the role of NK cells in TCE clearance, an adoptive transfer using donor NK cells from surgically-stressed and control mice was performed. Mice that had received surgically-stressed NK cells had significantly more metastasis three days after tumor cell injection. Furthermore, low-molecular-weight heparin (LMWH) was not able to attenuate metastasis in NK cell-depleted surgically stressed mice [57]. In addition, various other studies have also shown that heparin can reduce metastatic formation in murine models [62,63,64]. In patients with solid malignancies, prophylactic or therapeutic doses of LMWH in combination with standard therapy has proven to increase survival in some cancer patients [65,66,67]. In 1993 Kingston et al. analyzed the effects of the topoisomerase II inhibitor Razoxane and perioperative subcutaneous heparin on survival in a cohort of 603 colorectal cancer patients [66]. They observed a reduction in the risk of an event (recurrence or death) of 22% (95% CI: 3–37%) in heparin-treated patients. Additionally, von Templehoff et al. compared the effects of LMWH (Certoparin) versus unfractionated heparin on cancer survival in 160 breast and pelvic cancer patients and observed increased long-term survival among the LMWH-treated cohort [67]. Lazo-Langner et al. conducted a systematic review and meta-analysis of the effects of LMWH on cancer survival in 2007 [65]. They analyzed four studies for a total of 898 patients with solid tumors who were randomized to receive LMWH, placebo, or no intervention. The one-year mortality favoured patients receiving LMWH (risk ratio (RR) of death = 0.87 (95% CI: 0.77–0.99)). Moreover, patients with advanced disease (stages III and IV) also observed decreased mortality when administered LMWH (RR = 0.89 (95% CI: 0.80–0.99)) [65]. For these reasons, our group is conducting a phase 3 clinical trial whereby colorectal cancer surgery patients receive perioperative LMWH in an effort to reduce postoperative metastasis (PERIOP-01) [68]. 

### 3.2. The Release of Anti-Inflammatory Soluble Factors

Surgical stress results in an increase in suppressive soluble factors, such as anti-inflammatory cytokines and stress-related cortisol, prostaglandins, and catecholamines that may potentiate NK cell dysfunction. IL-6 and TGFβ are anti-inflammatory cytokines that have been shown to negatively regulate NK cell function [69,70,71,72]. IL-6 has both pro- and anti-inflammatory effects, but plays a predominantly anti-inflammatory role in the postoperative period [73] leading to reduced NK cell cytotoxicity through decreased perforin and granzyme production [74,75,76,77]. While there is a paucity of research into postoperative TGFβ, it is known to inhibit NK cell activation by downregulating the expression of activating NK cell receptors NKG2D, NKp30, and 2B4 [72,78,79,80,81]. Interestingly, IL-6 and TGFβ engage in a positive feedback loop resulting in heightened expression of each cytokine [82,83,84]. Stress-related factors with well-documented NK cell inhibitory functions, such as cortisol, prostaglandins, and catecholamines, are also increased postoperatively [85,86]. Strategies which aim to inhibit the release of these factors in response to surgical stress or antagonize their effects on NK cell function provide potential avenues for improving NK cell function in the perioperative period. Specifically, prostaglandin E2 (PGE2) exerts its effects directly on murine NK cells through four endogenous PGE2 receptors EP1-4 [87]. The small molecule inhibitor RQ-15986 has been shown to block EP4-mediated inhibition of NK cell function in vitro and in vivo [88]. Cyclooxygenase (COX)-2 is an enzyme that is responsible for the formation of prostaglandins from cell membrane phospholipids. Promising results in ongoing clinical studies where COX-2 inhibitors were used in conjunction with β-adrenergic receptor antagonists have provided evidence that pharmacological blockade of these suppressive soluble factors is responsible for the improved immune function and reduced metastasis [89,90,91,92]. Shaashua et al. investigated the use of propranolol (β-Blocker) and etolodac (COX-2 inhibitor) during the perioperative period in breast cancer patients and showed normalization of pre-surgical IL-6 and C-reactive protein levels, decreased epithelial-to-mesenchymal transition, and decreased tumor-infiltrating monocytes [91]. Currently, a multicentre randomized clinical trial to assess immunosuppression and cancer recurrence in colorectal cancer surgery patients treated with a β-adrenergic receptor antagonist and a COX-2 inhibitor is currently underway [90,93]. In addition to blocking the production of suppressive factors, other groups have explored the potential of using monoclonal antibodies or small molecules to antagonize NK cell receptors for soluble factors like IL-6, TGFβ, and PGE2 [76,88,94]. For example, the small molecule TGFβ receptor kinase inhibitor LY2157299 is able to mitigate the immunosuppressive tumor environment and preserve NK cytotoxic effector functions in vivo [94]. Finally, NK cell stimulating factors, such as IL-2, could be used in conjunction with these therapeutics in order to tip the balance and overcome the inhibitory signals from suppressive soluble factors in the postoperative environment.

### 3.3. The Expansion of Immunosuppressive Populations

#### 3.3.1. Myeloid-Derived Suppressor Cells

One of the greatest changes to occur postoperatively is the expansion of suppressive immune cell populations, specifically myeloid-derived suppressor cells (MDSCs) and regulatory T cells (T_regs_).

MDSCs are a heterogeneous population of immature immunoregulatory cells of the myeloid lineage. They can be either granulocytic/polymorphonuclear-, monocytic-, or early-stage MDSCs and have been reported to accumulate under various pathological conditions, such as cancer, burns, sepsis, infections, and following physical or surgical trauma [95,96,97,98,99,100,101,102]. These cells are characterized by myeloid specific markers, such as CD33 and CD11b, are negative for lineage markers (CD3, CD56, CD19), and they have little-to-no expression of HLA-DR in humans [103]. However, MDSCs are ultimately defined by their ability to suppress the activity of immune cells, such as T cells and NK cells [103].

Following cancer surgery, MDSCs rapidly expand in mice [101] and humans [104] and may play a major role in postoperative NK cell suppression. While multiple MDSC-mediated mechanisms of suppression have been described, the mechanism(s) by which MDSCs inhibit NK cell function in the postoperative period have yet to be elucidated. It is well described that after a traumatic injury or wounding, the body enters a state of emergency myelopoiesis whereby immature granulocytes and monocytes are rapidly generated and mobilized to sites of stress [105,106]. Recent studies from our group and others have reported that these myeloid cells possess an immunosuppressive phenotype after surgery and trauma [101,104,107]. In an experimental model of colorectal cancer, Xu et al. showed that surgery results in an increase in CT26 tumor growth with a concomitant increase in MDSCs and decrease in CXCL4 [108]. Interestingly, inoculating CXCL4 over-expressing CT26 tumors resulted in a decrease in MDSC infiltration in vivo and CXCL4 reduced MDSC migration in an in vitro transwell assay [108]. Previously, we have reported an increase in monocyte chemoattractants (MCP-1 and eotaxin-1) and a decrease in lymphocyte chemokines (6C-kine, IP10, and SDF-1) following surgery in mice [41]. These studies highlight the systemic rearrangement of immune cells and the accumulation of MDSCs in response to surgical stress.

MDSCs can suppress immune cell activity through many mechanisms, however here we will focus specifically on the documented mechanisms by which MDSCs suppress NK cells. Studies have shown that MDSCs can suppress NK cells in either a contact-dependent or independent manner. Sarhan et al. demonstrated that MDSCs isolated from patients with myelodysplastic syndrome suppress NK cell degranulation and IFNγ secretion through a contact-dependent mechanism mediated by TIGIT [97]. Anti-TIGIT blocking antibodies or separating cells via a transwell were sufficient to abrogate NK cell suppression [97]. Other groups have demonstrated the ability to prevent MDSC-NK cell suppression using TGFβ [102] and NKp30 [100] antibodies, or adenosine A2A receptor blockade [95]. Numerous groups have also described contact independent mechanisms of NK cell suppression by MDSCs. A hallmark of MDSCs is their upregulation of iNOS and/or arginase-1 which leads to the generation of nitric oxide (NO) or ornithine, respectively, through the catabolism of L-arginine [103]. A recent study by Stiff et al. showed that MDSCs can impair the Fc-receptor mediated (ADCC) and cytokine-producing effector functions of NK cells through NO production [109]. They were able to rescue NK cell ADCC by treating 4T1 tumor bearing mice with an iNOS inhibitor, NIL [109]. Furthermore, culturing patient MDSCs and NK cells in the presence of NIL restored NK cell ADCC lysis and IFNγ production. Interestingly, neutralizing antibodies against TGFβ and IL-10 were not effective. Lastly, nor-NOHA, the arginase-1 inhibitor, could improve NK cell ADCC in 4T1 bearing mice [109]. The increase in arginase-1 has been shown to be a major contributor to arginine depletion in the postoperative period [110]. In our murine studies, we have seen that inhibiting PDE5 with Sildenafil was effective in abrogating the suppressive effects of surgery-induced MDSCs on NK cells. In vivo administration of Sildenafil also led to reduced lung metastases following surgery in a murine model [101]. We speculate that this was due to the effect of PDE5 inhibition on MDSC arginase-1 expression. Our group and others have shown that PDE5 inhibitors can prevent MDSC suppression and decrease arginase-1 and iNOS expression [101,111]. We are currently testing the effects of Tadalafil in conjunction with influenza on MDSC function in the first phase 1b clinical trial of this strategy (PERIOP-04) [112]. These recent studies have shed light on the multifaceted ways in which MDSCs suppress NK cell function in addition to paving the way for therapeutic development. 

The first goal in the development of therapeutics to target MDSCs in the postoperative period is to concretely define the key mechanism(s) by which MDSCs induce NK cell dysfunction. Based on our current knowledge of MDSC-mediated NK cell suppression potential therapeutics would therefore likely benefit from targeting both contact-dependent and independent suppressive mechanisms. Monoclonal antibodies against TIGIT, TGFβ, and NKp30, or blockade of the adenosine A2A receptor would prevent the contact-dependent inhibition of NK cell effector functions. On the other hand, the pharmacologic inhibition of iNOS (1400W) or arginase-1 (norNOHA or CB-1158) activity would modulate soluble NO and arginine to improve NK cell activity [99,113,114,115,116,117]. There is currently an ongoing phase 1/2 clinical trial testing CB-1158 in conjunction with the PD-1 inhibitor Pembrolizumab in patients with solid tumors [118]. However, arginase-1 is important for wound healing [119,120] and therefore perioperative supplementation with arginine, perhaps in combination with other nutrients, such as omega-3 fatty acids, may prove to be more beneficial [121]. Heyland et al. completed a systematic review examining the relationship between enteral nutrition supplemented with immune-enhancing nutrients and infectious complications/ mortality rate in critically ill patients [122]. They reviewed 326 titles, abstracts, and articles and analyzed data from 22 randomized trials with a collective 2419 patients and found that immunonutrition was associated with lower infectious complications (pooled risk ratio (RR) = 0.66 (95% CI: 0.54–0.80)). Significantly, commercial formulas with high arginine content showed a more significant reduction in infectious complications with a trend toward a lower mortality rate and surgical patients treated with immunonutrition prior to surgery experienced significant benefit over critically ill patients [122]. Since MDSCs are a population of immature myeloid cells, inducing differentiation with all-trans retinoid acid (atRA) may reduce MDSC levels postoperatively [104,123]. Finally, identifying a specific marker for MDSCs that may be used for the development of antibody drug conjugates (ADCs) would allow for the selective depletion of the MDSCs, although this would be complicated by the heterogeneity of this suppressive cell population. 

#### 3.3.2. Regulatory T Cells

T_regs_ make up a subset of T cells with immunosuppressive properties defined by the expression of CD4 and CD25, as well as high levels of the forkhead box P3 (FoxP3) transcription factor [124,125]. The presence of T_regs_ in the tumor microenvironment and in circulation in cancer patients is associated with poor prognosis [125,126,127,128]. Initially studied for their role in suppressing cytotoxic T cells [125], T_regs_ have been increasingly associated with more widespread immune suppression, including IL-10-mediated suppression of NK cells [125,129]. In terms of surgical stress, and due to their close association with the tumor microenvironment, it was initially believed that T_regs_ would decrease following a major surgery [127,130]. When levels of circulating CD4^+^CD25^+^Foxp3^+^ cells were measured in patient PBMCs immediately following surgery on POD1 and were compared to preoperative levels, the data seemed to consistently agree with this hypothesis [104,124,127,130,131]. However, based on the association of T_regs_ and other biological stresses, such as septic shock, Saito et al. extended the blood collection to POD6 [124]. They found that not only did these regulatory subsets increase to levels higher than those observed preoperatively by POD6, the circulating levels of T_regs_ were also proportional to the level of surgical stress and were directly correlated to the invasiveness of the surgery [124], revealing them to be a novel marker of surgical stress. These T_regs_ were found to have higher expression levels of FoxP3, as well as increased PD-1 and CTLA-4 expression [104,132]. Furthermore, postoperative T_reg_ levels were positively correlated with cancer recurrence in both breast cancer and NSCLC, and may also prove to have prognostic value for other cancer types [104,131,132]. T_regs_ are able to functionally block NK cell activity and proliferation through various mechanisms, including cytokine release [129], the use of membrane-bound TGFβ [133], and out-competing the NK cells for Il-2 [129,134]. T_reg_ function and proliferation are promoted by catecholamines and prostaglandins [14,131,135,136,137], both of which increase in response to surgical stress [131]. In breast cancer patients receiving radical mastectomies, Zhou et al. found that the β-Blocker Propranolol, with or without a COX inhibitor, reduced the expansion of CD4^+^CD25^+^Foxp3^+^ cells in patient PBMCs on POD7, and that the T_regs_ in Propranolol-treated patients had reduced suppressive ability [131].

Various immunotherapies have been proposed to decrease T_regs_ found in cancer patients, and these may be effective in the perioperative period as well. Taking advantage of the contact-dependent mechanisms of suppression, such as T_reg_ upregulation of PD-1, CTLA-4, and membrane-bound TGFβ, means that checkpoint blockade could be effective in limiting the effects of T_regs_ on NK cell cytotoxicity. Targeting contact independent mechanisms of immunosuppression, such as blocking the activity of secreted cytokines, is also possible therapeutically. However, at this time blocking IL-10 production may prove to have too many side effects, due to its important role in controlling autoimmunity [138]. Since T_regs_ may be out-competing NK cells for IL-2, an important cytokine that regulates NK cell function, perioperative administration of IL-2 may be a viable treatment option to boost NK cell function. In fact, Klatte et al. and Böhm et al. investigated preoperative administration of IL-2 in renal cell carcinoma patients and showed improved immune function postoperatively [139,140]. Furthermore, Klatte et al. reported increased tumor-specific survival and progression-free survival in the IL-2 treated group at 1 and 5 years after surgery [139]. However, preoperative IL-2 may need to be employed with caution as Li et al. demonstrated that preoperative IL-2 therapy increased surgically-induced T_reg_ numbers and enhanced T_reg_ function in patients receiving radical mastectomies, and with melanoma or renal cancers [132,141]. Perhaps the most promising method of overcoming T_reg_-mediated immunosuppression postoperatively is to block T_reg_ proliferation and function, or to deplete this population from circulation. The combination of COX-2 inhibitors and β-Blockers have shown some efficacy in this regard [131]. Depletion of T_regs_, either through monoclonal antibodies or chemotherapeutics, is another option. In murine studies, CD25 depletion with or without low dose cyclophosphamide has consistently depleted regulatory T cells in vivo leading to improved therapeutics [142,143,144]. Recently, a number of clinical trials have looked at depleting T_regs_ in human cancer patients using either anti-CD25 therapies [145,146,147,148,149,150] or low dose metronomic administration of cyclophosphamide [151,152]. However, NK cells also express CD25 to form the high-affinity IL-2 receptor and this expression is critical to NK cell function [153]. Therefore, another T_reg_ marker that could be used for depletion may need to be identified.

Interestingly, MDSCs were found to interact with T_regs_ postoperatively [104]. Monocytic MDSCs (M-MDSCs) that underwent surgery-induced expansion interacted in a contact-dependent manner with CD4^+^ T cells, inducing the expansion of T_regs_ that displayed the surgical stress phenotype of high Foxp3 and high surface levels of PD-1. Furthermore, in a mouse model of surgical stress, when atRA was used perioperatively to treat mice, both MDSC and T_reg_ levels were reduced postoperatively and fewer lung nodules were observed [104]. Understanding the mechanism of surgical stress-induced T_reg_ expansion will allow us to determine which therapeutics to employ in the perioperative period to increase NK cell function. Finally, therapies targeting other immunosuppressive populations may be promising for their synergistic effect on T_regs_, as was observed with M-MDSCs [104]. 

## 4. Surgical Stress and NK Cell Biology

The complex postoperative immune suppressive environment presents multiple opportunities for therapeutic strategies aimed at reversing NK cell dysfunction. This postoperative milieu undoubtedly affects changes in NK cell biology which can serve as targets for immunotherapeutic development. While the suppression of NK cell effector functions persists for weeks [27,30,41], it is unknown whether dysfunctional NK cells are able to regain their lost functions or are instead replenished by newly differentiated, unimpaired bone-marrow-derived cells. For this reason, the armamentarium of therapeutics aimed at reversing NK cell-mediated postoperative metastasis must target activating/ inhibitory receptors and immune checkpoints, promote NK cell function through external stimuli, and deplete negative immunoregulatory NK cell populations in addition to supplementing endogenous NK cells with ex vivo and allogeneic NK cell populations. 

### 4.1. Targeting NK Cell Receptor Expression

If NK cell dysfunction is indeed a temporary state and functionality can be restored, then therapeutic strategies which enhance NK cell function, such as activating receptor agonists and immune checkpoint inhibitors may provide a therapeutic benefit. NK cell activity is controlled through combinatorial synergy of activating and inhibitory receptors to ensure specific and flexible response to external stimuli (as reviewed in [2,154]). A number of strategies which target these receptor families using soluble ligands, antibody crosslinking, and monoclonal antibodies have been investigated. Recently, Deng et al. have shown that the soluble form of the high-affinity tumor-associated NKG2D ligand MULT1 can enhance anti-tumor activity in murine NK cells [155]. This is presented in stark contrast to other studies, which describe down-modulation of NKG2D in response to sustained engagement with its soluble or membrane-bound ligands (as reviewed in [156]). These studies suggest that blocking prolonged NKG2D-ligand interactions, perhaps with monoclonal antibodies or small molecule inhibitors, may prevent the downregulation of this activating receptor. Furthermore, NKG2D ligand expression is regulated by DNA damage repair pathways, cytokines, and TLR signaling and can be controlled by pharmacological proteasome inhibitors and histone deacetylase inhibitors [157,158,159,160,161]. Upon investigation of NKp30 and a soluble form of its tumor-derived ligand B7-H6, Semeraro et al. suggest that NK cell activity may be improved by the neutralization of sB7-H6 in conjunction with activating NKp30 mAbs [162]. In addition to activating receptors, NK cells also express co-activating receptors, such as DNAM-1 and 2B4, which are crucial to NK cell function. Activation by monoclonal antibodies of 2B4 can stimulate IFNγ production and cytolytic activity in vitro [163], however, cytotoxicity was augmented if 2B4 and DNAM-1 were targeted together, suggesting that immunotherapeutic development should focus on preserving activating receptor expression, as well as targeting synergistic receptor pairs [2,163]. Positive results have also been observed in preclinical models using mAbs to target costimulatory receptors CD137 and CD27 [164,165,166,167,168,169]. Downregulation of stress-induced ligands by tumor cells is an important escape mechanism from NK cells [2,170]. Therefore, targeting activating receptors will not only enhance NK cell activity after surgery, but circumvent immune evasion mechanisms evolved in tumor cells.

As an alternative to targeting the repertoire of NK activating receptors, recent studies have suggested that blocking engagement/ signaling through inhibitory receptors can similarly improve NK cell function. This is a particularly attractive scenario given the clinical success of immune checkpoint blockade in restoring suppressed T cell functions. The surface receptors that inhibit NK cell function through the binding of constitutive self-signals are excellent targets for analogous checkpoint inhibition. The anti-KIR mAb Lirilumab is currently being used in clinical trials as a monotherapy or in combination with other checkpoint inhibitors against hematological and solid malignancies [2,171,172]. Gallois et al. showed that NK cell exhaustion was reversed in metastatic melanoma patients by blocking the activity of TIM-3 in vitro [173]. PD-1 expression has also been identified on NK cells and PD-1/PD-L1 blockade releases NK cell inhibition resulting in enhanced anti-tumor activity in various mouse models of cancer [174,175]. Furthermore, Benson et al. showed the enhanced function of human NK cells in vitro against multiple myeloma using the novel anti-PD-1 antibody CT-011 [176]. Anti-PD-1 therapies are also being investigated for the treatment of post-traumatic immunosuppression (PTI) in sepsis [177]. If postoperative NK cell functional suppression is mediated by an upregulation of inhibitory receptor expression and activity, anti-immune checkpoint therapeutics may be crucial in reversing this dysfunction. 

### 4.2. Promoters of NK Cell Function

NK cell activity can be modulated by more than just activating and inhibitory cell surface receptors. Depletion of arginine, a conditionally essential amino acid, has been shown to impair NK cell cytotoxic potential and the expanded MDSC population uses arginase-1 to deplete arginine postoperatively [178]. Interestingly, arginine supplementation is one of the treatments for PTI following surgery in non-cancer patients [179]. For this reason, we are conducting a clinical trial investigating a perioperative nutritional arginine supplement on NK cell activity postoperatively (PERIOP-02) [180]. NK cells also recognize virally infected cells, meaning that NK cell activity can be stimulated by the recognition of a virus, such as in the administration of oncolytic viruses (OVs) or vaccines. Tai et al. administered oncolytic parapoxvirus ovis (ORFV) and vaccinia virus (JX-594) perioperatively in a murine model of surgical stress and reversed NK cell suppression, as well as NK-dependent metastatic formation [27]. Furthermore, in the same murine model of surgical stress preoperative administration of influenza vaccination prevented postoperative NK cell dysfunction, as measured by tumor dissemination [101]. This vaccine modulated NK cell activity through dendritic cell-derived IFNα, as postoperative NK cells were still dysfunctional in IFNα receptor-deficient mice [101]. Thus, therapeutics should aim to combine direct targeting of changes in NK cell biology with external stimuli that may support or promote NK cell activity.

### 4.3. The Suppressive NK Cell

Changes in NK cell biology may also lead to the expansion of a population of immunosuppressive regulatory NK cells. Moore et al. first described the ability of NK cells to secrete the immunosuppressive cytokine IL-10 in 2001, and since then regulatory NK cells have been described in the settings of a variety of infections and cancers [6,181,182,183,184]. This phenotype switch, characterized by the ability to negatively regulate the innate and adaptive immune responses, appears to occur only in response to systemic and not local phenomena [182,184,185]. Jiang et al. describe an IL-10^+^ TGFβ^+^ NK cell population that is expanded during HIV infection and Perona et al. have characterized IL-10-secreting NK cells in response to systemic *T. gondii* and *L. monocytogenes* [182,185]. Furthermore, Terme et al. identified tumor-derived IL-18-induced Kit^+^CD11b^−^ NK cells that overexpress B7-H1/PD-L1 and promote tumor growth in two models of pulmonary metastasis [184]. Therefore, although the emergence of this population in the postoperative period has not been evaluated to date, it is possible that surgical stress induces the expansion of regulatory NK cells capable of suppressing both innate and adaptive immune responses. Finally, provided a regulatory NK cell population is in fact upregulated after surgery, a more complete identification of markers to define regulatory NK cells would be useful in the development of mAbs or ADCs to selectively inhibit or deplete this population postoperatively.

### 4.4. The Unresponsive NK Cell

The ability of therapeutic strategies targeting the activating or inhibitory receptors to reverse surgical stress-induced NK cell dysfunction is dependent upon whether NK cells can mount an appropriate cellular response to receptor engagement. This will not be the case if postoperative NK cells are functionally hyporesponsive or anergic. If surgically-stressed NK cells are incapable of regaining appropriate effector functions and instead have become anergic, therapies may include either induction of bone marrow progenitor proliferation (for new NK cell production) or adoptive cell transfer using autologous, allogeneic, or genetically engineered NK cell populations, in combination with ex vivo cultivation and in vivo cytokine therapies. NK cell differentiation from HSCs in the bone marrow has been well characterized and is controlled by various cytokines, including fms-like tyrosine kinase 3 ligand (FL), kit ligand (KL), IL-3, IL-12, IL-18, and common-γ chain family cytokines [186]. New NK cells produced from the bone marrow in the postoperative period may not exhibit the functional suppression displayed by mature NK cells present in the periphery during surgical stress. Zheng et al. present a manufacturing scheme for “off-the-shelf” universal KIR^−^ NK cells derived from induced pluripotent stem cells (iPSCs) which could be used postoperatively to deliver NK cells with intact effector functions [187]. Due to the innate ability of NK cells to recognize transformed cells, the adoptive transfer of NK cells, whether patient or donor-derived, has been investigated to treat a plethora of malignancies, including breast cancer, lymphoma, colorectal cancer, and melanoma [188]. However, long-term expansion protocols are still under development in an effort to produce clinical-grade NK cells [188]. Areas of importance include the source of the NK cells, cytokine stimulation, and cell culture medium in order to produce clinically relevant NK cell numbers with good purity, viability, and uncompromised anti-tumor activity [188,189]. Possible sources of NK cells include isolation from peripheral blood mononuclear cells (PBMCs) by apheresis or ficoll separation, stimulation, and differentiation from HSCs or iPSCs, or NK cell lines, with NK92s being the most widely studied. This isolation would be followed by NK cell expansion using feeder cells, stimulant cytokines, or both [187,188,190,191,192,193,194,195,196,197,198,199,200,201,202,203,204]. Numerous cytokines have been investigated for this purpose, including IL-2, IL-15, IL-21, IL-12, and IL-18 [189,195,205,206,207]. Due to the short half-life of IL-2 in serum (10 min), Nagashima et al. engineered NK cells to produce IL-2 resulting in a constant supply of IL-2 in vivo [208]. NK cells can also be genetically engineered to express chimeric antigen receptors (so-called “CAR-NKs”) to specifically target tumor antigens with less toxicity than CAR-T cells [209]. Thus, adoptive NK cell transfer using ex vivo expanded and activated genetically engineered NK cells could not only circumvent surgical stress-induced NK cell dysfunction, thereby preventing cancer recurrence, but could also lead to the effective targeting of residual cancer cells postoperatively. There are, however, questions about the practicality and feasibility of this type of treatment for surgery patients.

## 5. Summary and Where to Go from Here

**Figure 2:**
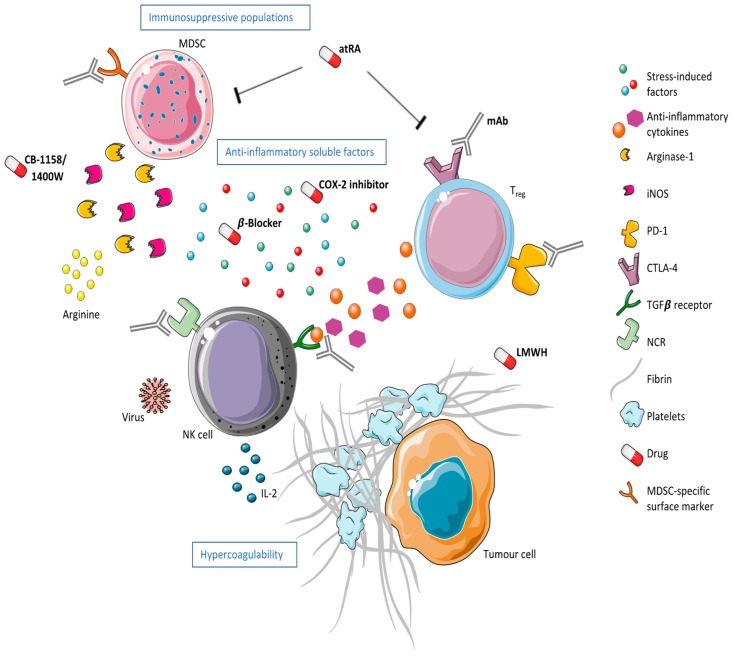
A suppressive environment alters NK cell biology in the postoperative period. A hypercoagulable state physically sequesters tumor cells from NK cells while soluble factors, such as anti-inflammatory cytokines and stress-related factors, as well as immunosuppressive MDSC and T_reg_ populations suppress NK cell activity postoperatively. This figure outlines potential therapeutics targeting extracellular and intracellular changes as a result of surgical stress. Low-molecular-weight heparin (LMWH) can inhibit P-selectin-mediated adhesion of activated platelets to tumor cells, thereby reducing tumor cell emboli. Pharmacological therapeutics such as CB-1158 or 1400 W could be used to inhibit arginase-1 or iNOS, MDSC-derived enzymes responsible for increased nitric oxide (NO) and reduced arginine postoperatively. COX-2 inhibitors could be used in conjunction with β-Blockers to inhibit the formation and release of stress-induced prostaglandins. All-trans retinoid acid (atRA) could be used to induce MDSC differentiation and/or deplete T_regs_. Monoclonal antibodies (mAb) could be employed to inhibit or activate receptors on the surface of immune cells, including NK cells, MDSCs, and T_regs_, in order to modulate their activity. Viral vaccines or oncolytic viruses could be used to promote NK cell activity against micrometastases. Finally, the administration of perioperative cytokines, such as IL-2, could boost NK cell function in the postoperative period.

Natural Killer cells are innate lymphocytes with cytotoxic, cytokine-secreting, and apoptosis-inducing effector functions that play a critical role in the anti-tumor immune response. Although tumor removal is a necessary intervention in the treatment of solid malignancies, surgery is associated with increased metastasis and cancer recurrence. Suppression of the cellular immune response, specifically NK cells, is responsible for this phenomenon. The use of NK-boosting therapies, such as IL-2 or IFNα, in the perioperative period has shown promising results. However, there is untapped potential in the use of immunotherapies to reverse or prevent surgical stress-induced NK cell dysfunction. Postoperative hypercoagulability, the release of suppressive soluble factors, and the expansion of suppressive cell populations create an immunosuppressive environment under which NK cells cannot exert their effector functions (Figure 2). This results in altered NK cell biology: Downregulation of activating receptors/upregulation of inhibitory receptors and immune checkpoints, induction of anergy resulting in an inability to respond to extracellular signals, or phenotype switching/expansion of immunosuppressive NK cell populations. Individually, each of these potential mechanisms of NK cell dysfunction provides an opportunity for immunotherapeutic intervention. However, NK cell dysfunction in the postoperative period is more likely a complex combination of an immunosuppressive environment and changes in NK cell biology. Perioperative combination therapy in cancer surgery patients will therefore be the most promising avenue for the prevention of cancer recurrence. We are currently conducting a phase 1b clinical trial in which a carefully selected promising perioperative immunomodulatory regimen is aimed at preventing MDSC suppression of NK cell function using Tadalafil while simultaneously activating NK cells using influenza vaccination to safely and effectively reverse the effects of surgical stress on NK cell function (PERIOP-04) [112]. The future of immunotherapeutics in cancer and specifically in modulating immunosuppression in the postoperative period should focus on combination therapies that target not only NK cell biology, but also the immunosuppressive environment induced by surgical stress to reduce rates of metastasis and cancer recurrence for all surgical oncology patients.
